# Using animal-derived constituents in anaesthesia and surgery: the case for disclosing to patients

**DOI:** 10.1186/s12910-019-0351-4

**Published:** 2019-02-18

**Authors:** Daniel Rodger, Bruce P. Blackshaw

**Affiliations:** 1 Department of Allied Health Sciences, School of Health and Social Care, London South Bank University, 103 Borough Road, London, SE1 0AA England; 2 Department of Philosophy, School of Philosophy, Theology and Religion, University of Birmingham, Birmingham, England

**Keywords:** Animal-derived products, Informed consent, Vegetarianism, Veganism, Principlism, Montgomery, Paternalism, Anaesthesia, Surgery, Dressings

## Abstract

**Background:**

Animal-derived constituents are frequently used in anaesthesia and surgery, and patients are seldom informed of this. This is problematic for a growing minority of patients who may have religious or secular concerns about their use in their care. It is not currently common practice to inform patients about the use of animal-derived constituents, yet what little empirical data does exist indicates that many patients want the opportunity to give their informed consent.

**Discussion:**

First we review the nature and scale of the problem by looking at the groups who may have concerns about the use of animal-derived constituents in their care. We then summarise some of the products used in anaesthesia and surgery that can contain such constituents, such as anaesthetic drugs, surgical implants and dressings. Finally, we explore the problem of animal-derived constituents and consent using Beauchamp and Childress’ four principles approach, examining issues of autonomy, beneficence, nonmaleficence and justice.

**Summary:**

Disclosing the use of animal-derived constituents in anaesthesia and surgery is warranted under Beauchamp and Childress’ four principles approach to the problem. Although there exist systemic and practical challenges to implementing this in practice, the ethical case for doing so is strong. The Montgomery ruling presents additional legal reason for disclosure because it entails that patients must be made aware of risks associated with their treatment that *they* attach significance to.

## Background

Providing healthcare in a culturally diverse society can involve a number of unique challenges and compromises. The problem of animal-derived products in healthcare has received some attention in the literature [1–3]. However, very few clinicians remain aware of the scale of the problem, and even fewer have considered how it could be navigated in a way that respects the rights and interests of patients. When so many individuals and groups differ on fundamental concepts, what constitutes care for one patient could be interpreted as harm by another. Some of these fundamental disagreements surround the ethical permissibility of products utilised in healthcare that contain an animal-derived constituent.

Patients with certain religious and secular beliefs may express concerns about the use of animal-derived constituents in their care, and in some cases, this can result in a patient refusing certain kinds of treatment [1, 4–6]. The area of perioperative care is notably problematic because of the broad range of products employed that may contain animal-derived constituents, such as anaesthetic drugs, surgical implants and dressings. An increasing number of individuals would like to and may even expect to be informed of any animal-derived constituents that could be used in their care.

The United Kingdom (UK) represents a diverse and multicultural population who hold a variety of religious and secular beliefs about the permissibility of using animals for food, clothing, research or medical products, and this population is increasing. Between the 2001 and 2011 censuses, the Muslim population in England and Wales increased by 1.2 million, and from 3% to 5% of the total population [7]. For Muslims, products containing animal-derived constituents are unlikely to be *halal*—compatible with Islamic dietary laws. Furthermore, research commissioned by The Vegan Society found that about 1% or over 500,000 of the over-15 UK population now follow a vegan diet which amounts to more than a 300% increase since 2006—a significant proportion of who avoid non-dietary animal products [8]. This steep increase in both the Muslim and vegan populations implies that the number of individuals who might object to the use of animal-derived products used in healthcare is growing rapidly. This raises some significant ethical and practical issues for clinicians, the most pertinent being deciding what information should or should not be disclosed to patients about the products being used to treat them.

The importance of disclosure has been recently highlighted by the Montgomery v Lanarkshire Health Board [2015] case, which has transformed UK law on informed consent [9]. With regard to informing the patient of any material risks involved in their treatment, the Montgomery ruling shifts the emphasis from what clinicians would agree is reasonable, to what a reasonable patient would expect to know. The ruling means that clinicians must communicate relevant information to a patient, even if its disclosure could lead the patient to make a choice that may not be in their best interest [10]. Patients are entitled to make decisions that a clinician disagrees with, unless they lack capacity to make an informed choice [11]—even if a clinician is concerned that disclosing certain information could cause psychological harm or lead a patient to refuse life-saving treatment [12].

In this article, we explore which patients may wish to be informed about the use of animal-derived constituents in their anaesthetic and surgical care, and then describe some of the products that may contain them. We then consider some of the ethical issues associated with using these products in anaesthesia and surgery. Employing Beauchamp and Childress’ influential four principles of bioethics—autonomy, beneficence, nonmaleficence and justice—we examine the case for disclosing this information to patients and the potential challenges of doing so [13].

### Who has concerns about the use of animal-derived constituents?

In 2017, the Vegan Society sent a letter to the UK’s Secretary of State for Health, detailing their concerns about the use of animal-derived constituents in healthcare, and the current lack of transparency on the labelling of medicines [14]. Understandably, the growing number of vegetarians and vegans want to know if the drugs they might be prescribed contain animal-derived constituents. Individuals are increasingly restricting their consumption and use of animal-derived products for a number of reasons: perceived health benefits, environmental concerns, ethical reasons such as promoting animal welfare, and objections to the intentional killing of non-human animals [15]. It has been estimated that between 3 and 7% of the UK population are vegetarian [16].

Importantly, not all vegetarians would necessarily object to the use of drugs, dressings or implants that have animal-derived constituents. Merely being vegetarian does not necessarily entail objecting to the use of animal-derived products, since some reasons for being vegetarian may not extend beyond dietary preferences [2]. This is in contrast with vegans who typically will not eat or use dairy products, eggs or other animal-derived products. Vegans are much more likely to have concerns about any animal-derived products that may be used in their care, and will want to be fully informed. Many will wish to avoid animal-derived products as far as possible and opt for an alternative, should one exist. Charlotte Houltram, a vegan anaesthetist, has stated that achieving an animal-derived product free anaesthetic can be practicable and offers helpful guidance on how to do so [17]. Houltram notes that because all drugs currently in use in the UK have been tested on animals during their development, none of them can be considered to be fully vegan. Consequently, their use will always involve a degree of compromise.

There is also a more sizeable population who potentially may object to the use of animal-derived products in anaesthesia and surgery for religious reasons. In one survey of 13 representative religious leaders, 10 (77%) expressed concerns about the use of biological products—including those with animal-derived constituents—in healthcare and thought that informed consent should be obtained from patients [18]. A subsequent study by Eriksson et al. explored which religious groups object to the use of drugs, implants and dressings containing animal-derived constituents [19]. It was discovered that a number of the world’s largest religions objected to the use of some animal-derived products. For example, Muslims—Sunni and Shiite—in the study objected to the use of any porcine-containing products. In Islam, pork and its by-products are considered ‘haram’ which means forbidden and so for some Muslims the use of porcine-containing products is impermissible, even if the alternative non-porcine containing product—should one exist—meant that healing and recovery was delayed or it was more expensive [18, 20]. For some Muslims, what would be unlawful to consume can also be considered unlawful in the context of healthcare [21]. Nevertheless, Islam is not homogenous and there remains no definitive consensus regarding the use of products that contain an animal-derived constituent [20, 22]. Individual Muslims may therefore have differing beliefs and ways to navigate such conflicts should be considered [21].

Hindus from one of the major traditions known as Vaishnavism objected to the use of drugs, implants and dressings that contain porcine and bovine constituents [19]. This view may not necessarily be representative of all of the traditions within Hinduism, although the majority are vegetarians and avoid egg. However, the use of any animal-derived products may be problematic for many Hindus, with many categorically rejecting their use [1, 23]. The Christian, Jewish and Buddhist leaders contacted by Eriksson et al. had no objections to any animal-derived products [19]. However, all of the religious groups that objected to the routine use of animal-derived products stated that they would be permissible providing (1) there was no viable alternative and (2) they were required for emergency treatment [19]. However, what is classed as emergency treatment can be ambiguous. Some kinds of surgical procedure containing animal-derived products may not be life-saving, but they may have a substantial positive impact on a patient’s long-term quality of life and general wellbeing.

Neither religious or secular groups are homogeneous in their views on the use of animal-derived products used in their care. Consequently, there is considerable diversity of opinion, and membership of a particular group does not necessarily dictate an individual’s convictions. The increase in the number of religious and secular individuals who might be concerned about the use of animal-derived constituents indicates that this is an issue healthcare professionals can no longer afford to ignore.

### Drugs, dressings and implants containing animal-derived constituents

A number of products used in anaesthesia and surgery contain animal-derived constituents that some patients may object to, were they to know. What is particularly problematic is that patients who may have concerns are rarely informed that some of the products utilised in their care may contain animal-derived constituents. These include a number of anaesthetic drugs, surgical implants and dressings. For instance, some drugs contain animal-derived excipients (substances that do not contribute to the therapeutic action), which can make up 90% of the formulation [24]. These cannot always be easily identified because there is currently no legal requirement for them to be included on the packaging.

In one study it was discovered that of the 100 most prescribed drugs in primary care in the UK, 74 contained an animal-derived ingredient (most commonly bovine and porcine sources) [3]. This highlights the scale of the problem for religious and secular patients who may object to the use of products with animal-derived constituents. For instance, amoxicillin capsules can be prescribed postoperatively before discharge, yet they contain porcine or bovine sourced gelatin, which is widely used to encapsulate medication [25]. Some capsules can be opened to avoid ingesting the gelatin, but this is not always advisable, unless clearly stated on the patient information leaflet. However, amoxicillin in oral suspension is suitable for vegans and is one alternative for patients who would prefer to minimise their use of animal-derived constituents.

The most commonly used induction agent in anaesthesia—Propofol—contains purified egg phosphatide, which vegans would want to avoid, and as it is likely the eggs used are not free range, many vegetarians would similarly find its use problematic [17, 26]. Avoiding Propofol is not an insurmountable problem and could be managed relatively easily by an anaesthetist but it will depend on the kind of induction that is clinically indicated. There is a formulation of Propofol called Cleofol®, which is manufactured in India and does not contain any egg phosphatide or other animal-derived constituents [27]. However, its use has been associated with excessive pain on injection [28]. This may not be sufficient to rule out the use of Cleofol® as a viable alternative for patients who wish to avoid animal-derived constituents. After all, a well known side-effect of Propofol is pain on injection, which occurs in 60% of untreated patients; pain on injection can be mitigated by using an antecubital vein or pre-treatment with intravenous (IV) Lidocaine [29]. Moreover, Houltram suggests that patients could avoid Propofol by opting for local or regional anaesthesia, or where appropriate by using an induction agent that does not contain any animal-derived constituents such as Thiopentone, or by having a gas rather than IV induction [17]. She does note that having a gas induction may be contraindicated in some cases.

Hydrocolloid dressings can contain porcine-derived gelatin and are routinely used for a number of different types of hip and knee surgery [30, 31]. Most commonly in the form of DuoDERM® which can also be used to help secure endotracheal tubes (ETT) or nasopharyngeal airways, and protect skin integrity in neonates and infants [32, 33]. In a study by Enoch et al. published in 2005, it was discovered that very few healthcare professionals were aware of which commonly used dressings contained biological material, and were therefore not necessarily in a position to inform patients [18]. Enoch et al. subsequently recommended that hospitals, higher education institutions and product manufacturers should take immediate action to ensure that healthcare professionals are educated about the biological products they frequently use [18]. The lack of education and easily available information creates a barrier to informing patients who might object to the use of certain biological products, including those with animal-derived constituents. However, there is little evidence to suggest that much has changed in more than a decade.

There are also a number of surgical products or implants that could also be objectionable to a number of patients. These include composite and biological mesh commonly used to repair the abdominal wall during hernia surgery and in some types of colorectal surgery [4, 34]. They frequently use ingredients from porcine or bovine sources, and this information is not always easily accessible. Biopolymer sutures can also contain animal-derived constituents, including those from ovine and bovine sources [35]. Other types of surgical products that can contain animal-derived constituents include orthopaedic spacers, heart valves and haemostasis matrix [19].

This brief summary of animal-derived products used in anaesthesia and surgery shows that clinicians are routinely using drugs, dressings and surgical implants that a sizable minority of patients in the UK may have a material interest in being informed of. We are not suggesting that there are any easy answers: we are keenly aware of the existing systemic obstructions, and some of the reasons clinicians may be hesitant to acknowledge that this practice could be ethically problematic enough to warrant any serious concern. The health service is already over-stretched, under-resourced, understaffed and many clinicians are already struggling to provide patient care under those existing constraints. Nevertheless, the General Medical Council’s guidance on personal beliefs and medical practice state that when ‘…assessing what is of overall benefit to adult patients, you must take into account their cultural, religious or other beliefs and values’ [36]. In principle, it does not seem unreasonable to us to conclude that this guidance implies that patients should be informed about the use of animal-derived products in their care. Furthermore, surgical (and medical) inpatients are routinely asked about their dietary requirements; there is no expectation that they must self-declare that they require vegan, halal or kosher food. It seems inconsistent to go to the effort and expense of respecting patient’s religious or secular dietary choices and then use products in their care that many of them may feel as strongly about. Informing patients can therefore demonstrate a more holistic approach to their religious or secular beliefs, rather than maintaining what could be interpreted as a paternalistic approach to the issue.

### Applying the four principles approach

We indicated that Beauchamp and Childress’ four principles approach will be used to examine the issue of disclosure of animal-derived products in anaesthesia and surgery. We will consider what each principle informs us about the problem, and attempt to weigh any competing concerns that are uncovered. Beauchamp and Childress recommend a process of deliberation to decide the relative weights of each norm in this context, so that balancing judgements can be made if conflicts between principles are found when they are applied [13]. Beauchamp and Childress offer some practical conditions for restricting balancing to prevent the process from being too open-ended, such as requiring that any infringement of a principle is the least possible infringement with no better alternative, and has realistic prospects of achieving the objective [13].

### Autonomy

The first principle is respect for autonomy: acknowledging a patient’s right to make choices based on their personal values and beliefs, the paradigmatic example being obtaining their informed consent before treatment. One of the key elements of informed consent is disclosure of relevant information, including ‘those facts or descriptions that patients or subjects usually consider material in deciding to refuse or consent to the proposed intervention or research’ [13]. Typically, disclosure might involve factors such as risks and benefits of a procedure, but Beauchamp and Childress also suggest disclosure should be ideally tailored to ‘the specific informational needs of the individual person’, needs which might include ‘unconventional beliefs’ [13]. Although only a small minority of the population is vegan, there are also significant numbers of people who avoid certain animal-derived foods for religious reasons, and there is widespread acceptance of disclosure of the use of animal-derived products in the food industry. As already noted, it is common practice in UK hospitals to request inpatient preferences for their dietary requirements, religious or otherwise.

It seems reasonable to inform patients of the use of animal-derived products in their treatment, even if it is not possible to avoid their use. Although the recent Montgomery v Lanarkshire Health Board [2015] case concerned clinical risks that could lead to patients being harmed, it has established a precedent in the UK that the patient’s autonomy must be respected—the patient must be made aware of material risks involved in a recommended treatment as well as reasonable alternatives [9]. A risk is defined as material if the patient is likely to attach significance to this risk. Although in the context of the Montgomery case this refers to risks of the medical procedure, the legal implications could be broader: arguably, some patients could be harmed by being treated with animal-derived products if they have strong moral or religious concerns and they later learn this has occurred. It is not for clinicians to assess the significance of this risk on their patient’s behalf. Autonomy as understood by Beauchamp and Childress entails that autonomous agents have the right to act according to their values and beliefs [13]. If a clinician, for instance, knows that their patient is Hindu, Muslim or vegan and fails to disclose the use of known animal-derived products in their care, they may be disrespecting their patient’s autonomy.

Do patients want to know this information? Respect for patient autonomy entails a presumption that they do, and this has been reinforced by the Montgomery ruling. One study from the United States (US) found that 84% of patients sampled were unaware that over 1000 medications contain animal-derived constituents, and 63% wanted their physicians to inform them about medications that contained these products [37]. This provides empirical support for the claim that many patients would prefer to be informed about the use of animal-derived constituents in their care. Interestingly, 70% of physicians believed that providing their patients with this information was important, although acknowledging this seldom lead to any changes in practice [37].

The available evidence therefore indicates that a significant proportion of patients may want this information disclosed to them, and given the importance of respect for patient autonomy, this implies clinicians should not presume patients are uninterested but rather ascertain if the use of animal-derived constituents is an issue for them. If so, they should, as far as possible, disclose the use of known animal-derived constituents in the patient’s care to ensure their concerns are adequately catered for.

### Nonmaleficence

The second principle is nonmaleficence, or the obligation not to inflict harm. According to Beauchamp and Childress, harm is a thwarting, defeating, or setting back of some party’s interests, and it is a prima facie principle that harmful actions must be sufficiently justified [13]. While this commonly refers to physical harm, it also encompasses psychological harms, which describes harms that can damage someone’s psychological well-being. As we indicated earlier, patients who may be concerned about the use of animal-derived products in their care may consider that they have been psychologically harmed by not having that information disclosed, and later learning they have been treated with animal-derived products without their consent.

Clinicians may consider such objections unconventional and obstructive, nevertheless, harm can be very subjective and individualistic, and a clinician cannot presume any resulting psychological harms would be trivial. Some vegetarians have described feeling *defiled* at the thought of accidentally eating meat—anecdotal accounts describe such an experience as ‘upsetting’—and so it is possible that failing to convey the use of an animal-derived product could cause some degree of psychological harm [38, 39]. Of course, there may not be an alternative available, and it might be that a clinician considers it is justified in avoiding disclosing such information on the grounds that the patient may be harmed if they refuse treatment.

Potential harm from the patient’s refusal to accept treatment does not, however, seem sufficient grounds to trump the combination of respect for patient autonomy and the potential of later psychological harm. Failure to disclose the information on the basis that the patient may make a decision that may not be in their interest is also not compatible with the Montgomery ruling. Unless the patient lacks capacity to make an informed choice, they are entitled to refuse treatment if they believe that doing so would harm them, and even if doing so could lead to additional harms. We have already established that some patients would object to the use of animal-derived products on religious or secular grounds, and it seems likely they may be distressed to learn that products with these constituents were used in their treatment, without their consent. Previous studies have identified that most religions make a provision for the use of animal-derived product in an emergency—such as life-prolonging surgery—or when all options for a clinically appropriate alternative have been exhausted [1, 19]. Nonmaleficence entails an obligation on clinicians to not impose unnecessary harms; disclosing this information and gaining informed consent would be one appropriate measure to avoid causing this kind of harm.

### Beneficence

The principle of beneficence is closely related to nonmaleficence—the requirement to contribute to the welfare of the patient (rather than harm them) by acting in their best interest. It is important to consider if disclosure of the use of animal-derived products in perioperative care will hinder patient welfare in some way. Obviously, the patient’s treatment will be intended to benefit them, and it is possible that disclosing this information could result in the patient refusing certain kinds of treatment. This is not just a theoretical concern, as we noted in our introduction and discussed in the previous section.

Sattar et al. describe four cases of medication non-adherence because of patients’ concerns about the use of certain animal-derived constituents that they believed to be forbidden by their religion [40]. The patients only stopped taking their medication upon finding out for themselves that it contained an animal-derived constituent. This led to either worsening symptoms or relapses of their illness, and therefore a violation of the principle of beneficence. Naively, withholding information might be thought to promote beneficence because of the risk that some patients might decline treatment, but as these examples show, some patients will investigate for themselves. Consequently, in these four cases of medication non-adherence, the cause was the *absence* of disclosure and any conversation about the contents of their medication prior to them being prescribed. All four patients were subsequently provided with medication that did not contain an animal-derived constituent and that satisfied their religious concerns. Beneficence in these cases would have been achieved by disclosing this information, obtaining informed consent and amending the medication in light of the patient’s religious beliefs.

A UK questionnaire-based study of a mixed ethnicity inner-city population found that 43% would avoid taking prescribed oral medication containing animal-derived ingredients, even if there was no alternative treatment [41]. However, only 20% of those who preferred vegetarian medication would ask the prescribing doctor if their medication contained animal-derived ingredients. In the perioperative context, this highlights the importance of communicating the possibility of animal-derived constituents being used to pre-empt these concerns. This reinforces our earlier point regarding the potential harm that may eventuate from the use of these constituents and the subsequent discovery of this by patients who have strong religious or secular concerns with their use.

There is still the issue of those patients who refuse treatment because of their convictions. What course of action should be taken in these situations? It seems that there is an obligation to ensure that alternative products are available where possible, at least for commonly used products. In addition, patient disclosure should include variations in risks and benefits incurred by using alternative products, and of course if a suitable alternative exists and is available. Alternatives may not be available in emergencies, but we have noted that religious groups and vegans may not object to the use of animal-derived constituents in these situations in the absence of alternatives. Clinicians could also suggest, where possible, that the patient discuss their concerns with a religious leader or secular authority. In instances where a patient still refuses treatment because of unavoidable animal-derived constituents, the patient’s wishes must be respected, provided they have capacity to make an informed choice.

### Justice

The final principle is justice, or the equitable distribution of goods—healthcare in this case. We do not have space to discuss different principles of distributive justice that could be employed, or whether there is a general right to healthcare and how it might be implemented. For our purposes, we will assume that all patients deserve a minimum standard of care, and the standard provided should be equivalent for each patient. In practice, healthcare resources are always finite, demands are high, and consequently some form of rationing and setting of priorities is always required. We can grant a prima facie obligation to provide groups with certain religious or moral convictions about animal-derived products with alternative products so they can receive equivalent care, but budgetary limitations may constrain what can be offered. Constraints include the availability and affordability of viable alternatives. Additionally, clinicians must be aware of these alternatives, and be able to provide an assessment of their comparative efficacy and risks. These constraints are considerable, and we must ask, to what extent should clinicians and budgets be required to cater for patients with these convictions? The answer will be dependent on each hospital’s budget, as well as the particular product involved—some alternatives may be considerably more expensive than the equivalent animal-derived product.

An additional consideration, raised by Newson, is that some synthetic alternatives may have greater efficacy than their equivalent [42]. This may mean that those patients with religious and moral convictions about the use of animal-derived constituents may receive more effective treatment than most patients, which seems unjust. The solution may be to provide the synthetic version to all patients, but if this incurs significantly increased costs, it may impact the provision of health services in other ways, and this may not be acceptable. Of course, some non-animal-derived drugs may well be less effective than their equivalents, but in these scenarios, the patient can be given the choice of either drug.

For synthetic alternatives that prove too expensive, there is another approach that could be considered. The minimum standard of care provided could exclude the provision of synthetic drugs except where they incur similar costs, and patients whose convictions require them to avoid animal-derived products could pay for these alternatives themselves, either via a private health insurance policy designed for the purpose, or directly. If such a policy is made widely known, over time those who hold such convictions could ensure they make provision for this.

What are the implications for disclosure? If, as a matter of policy, all clinicians were required to disclose the use of animal-derived products to their patients, demand for alternatives would increase and ultimately costs of these alternatives should decrease. In many instances, synthetic alternatives could gradually replace certain animal-derived products for all patients. This would, over time, make healthcare more accessible for those who hold convictions about the use of animal-derived products. There are, however, a number of complications that could ensue and which we now briefly explore.

### Challenges of disclosure

We have sought here to make the ethical case for disclosure by considering the problem using Beauchamp and Childress’ four principles approach. We will now review some of the challenges that implementing it might generate in anaesthesia and surgery and beyond. It is primarily problematic for a very practical reason: very few clinicians know whether or not the drugs, surgical products and dressings they use contain any animal-derived constituents. Additionally, as Tatham and Patel note, ‘information about animal derived products in medicines is difficult to obtain, unclear, inconsistently reported, and sometimes incorrect’ [3]. Even clearer labelling, however, is not sufficient, as a comprehensive knowledge is also required of what acceptable alternatives—should they exist—to each drug or product are available, including efficacy and comparative risks.

These types of systemic obstructions mean that clinicians are limited in what they can actually promise a patient if they request that they want their care to be free of animal-derived constituents. Clinicians can undertake to minimise their use, but will need to communicate that insufficient information exists regarding the constituents in every product they are using, and so it is possible that some products may still contain animal-derived constituents. Any more than this may not be practicable until there has been significant changes in product labelling. In some cases, surgeons will know that a product—such as a biological surgical mesh containing porcine tissue—contains a constituent that a patient may object to, and here we have established that patient concerns should be preempted and informed consent should be obtained. In the context of anaesthesia, it may be possible for an anaesthetist to provide an ‘animal product free anaesthetic’ for patients, providing as we have already discussed, that this is compatible with the kind of induction that is clinically indicated [17]. This does still raise additional clinical concerns: it is conceivable that in trying to meet the demands of patients who want an ‘animal product free anaesthetic’ that this could lead to an increased risk of anaesthetic complications. For instance, the use of Thiopentone in obstetrics has been associated with increased drug errors, and it is relatively unfamiliar to the newer generation of anaesthetists who most frequently use Propofol to induce patients [43].

The task of disclosure would be far easier if a legal requirement was introduced forcing the declaration of inactive animal-derived constituents. A database of medicines licensed for use in the UK already exists: the electronic Medicines Compendium (eMC) [44]. The eMC contains a Summary of Product Characteristics (SmPC) for each medicine, which is comprised of detailed information supplied by pharmaceutical companies, including active and inactive constituents. This information is checked and approved by the UK or European government agencies which licence medicines. It would be straightforward to augment the existing data to indicate what constituents are animal-derived, and this would be a significant step forward, at least for medicines. However, dressings and surgical implants are not included in this compendium. Additionally, knowledge of acceptable alternatives—should they exist—to each drug or product are required, including efficacy and comparative risks. It seems feasible to include this information, although this would be a more complex task. This would be broadly useful in healthcare far beyond anaesthesia and surgery, and indeed, the case for disclosure presented here is likewise widely applicable.

A long-term solution, suggested by Tatham and Patel, would be to eliminate all animal-derived constituents from all drugs and medical products [3]. This may seem unlikely in the short-term, given the number of products that would require modification or replacement, and the potential costs that would ensue. There are also no incentives for medical and pharmaceutical companies to even consider this development. However, were disclosure to be advocated, clinicians and patients could exert pressure on companies to develop products that would alleviate their religious or secular concerns. Finally, an important component of successful disclosure is improved education among healthcare professionals regarding the use of animal-derived constituents. Higher education institutions must consider their responsibility to educate healthcare professionals about the ethical and legal issues surrounding the use of animal-derived products in healthcare.

## Conclusion

The use of animal derived constituents in anaesthesia and surgery is of increasing interest for a growing minority of patients, many of whom have religious or secular concerns about their use. There has been a significant rise in the Muslim and vegan populations in the UK, and we have argued that existing research supports the contention that many of them believe that they should be given the opportunity to provide informed consent. Clinicians should expect to be increasingly confronted by patients who wish to know if any products used in their care contain animal-derived constituents, and whether they can be avoided. Clinicians must therefore consider how they will manage the concerns of such patients.

We have applied Beauchamp and Childress’ four principles to this issue, and conclude that they provide a strong case for disclosing the use of animal-derived constituents to patients in anaesthesia and surgery, as well as other areas of healthcare. The importance of patient autonomy provides the strongest argument for disclosure. The Montgomery ruling, while a legal motivation rather than an ethical one, also emphasises the importance of patient expectations about disclosure, and we have established that for many patients, the use of animal-derived constituents is a significant issue.

We acknowledge that full disclosure faces some practical difficulties at present: there are a number of systemic obstructions that will need to be dealt with which will require significant changes to the law, product development, education and clinical practice. Nevertheless, our analysis strongly supports the contention that informed consent should be gained from patients when animal-derived constituents will be used in their care, and that alternatives should be sought where available and practicable.
